# CSF galanin and noradrenaline downregulation by psilocybin therapy in major depressive disorder

**DOI:** 10.1038/s41386-026-02490-3

**Published:** 2026-07-07

**Authors:** Wojciech Paslawski, Daniel Doyon, Carl Johan Ekman, Hampus Yngwe, Dejan Mamula, Justyna Zareba-Paslawska, Maria Beckman, Zhi-Qing David Xu, Tomas Hökfelt, Mikael Tiger, Johan Lundberg, Per Svenningsson

**Affiliations:** 1 Translational Neuropharmacology, Department of Clinical Neuroscience, Karolinska Institutet, Stockholm, Sweden; 2 Centre for Psychiatry Research, Department of Clinical Neuroscience, Karolinska Institutet, and Stockholm Health Care Services, Region Stockholm, Stockholm, Sweden; 3 Departments of Neurobiology and Pathology, Capital Medical University, Beijing, China; 4 Department of Neuroscience, Karolinska Institutet, Stockholm, Sweden

**Keywords:** Outcomes research, Depression

## Abstract

Psilocybin is a rapid-acting antidepressant, but its mechanism of action in major depressive disorder remains unclear. In this secondary analysis of randomized, placebo-controlled trial with multimodal CSF and blood biomarker measurements, psilocybin selectively reduced CSF galanin and noradrenaline, implicating that normalization of these co-transmitters is a key pharmacodynamic signature.

## Introduction

Major depressive disorder (MDD) is one of the leading causes of disability worldwide [1]. Despite decades of pharmacological research, up to two-thirds of the MDD patients fail to achieve remission with first-line treatment, and up to one-third meet criteria for treatment-resistant depression (TRD) [2]. This therapeutic shortcoming reflects an incomplete mechanistic understanding of MDD pathophysiology and underscores the urgent need for novel treatment strategies accompanied by robust, mechanistically informative biomarkers.

Psilocybin has emerged as one of the most promising candidates for rapid-acting antidepressant treatment. As a preferential 5-HT2A receptor agonist, acting via its active metabolite psilocin, psilocybin initiates a cascade of neurobiological effects culminating in rapid, substantial, and sustained reductions in depressive symptoms demonstrated in multiple randomized controlled trials [3–5].

The present study is a secondary analysis designed to characterize the biological effects of psilocybin-assisted therapy in MDD through a randomized, placebo-controlled design incorporating a comprehensive, multi-modal biomarker strategy [6]. Cerebrospinal fluid (CSF) measurement of monoamine neurotransmitters and their metabolites was included to monitor the dysregulation characteristic of MDD and their modulation by psilocybin. A targeted cytokine panel was incorporated to assess the neuroinflammatory dimension of MDD [7] and psilocybin’s proposed immunomodulatory effects, given consistent evidence that psilocybin augments anti-inflammatory signaling [8]. BDNF was measured as a canonical index of neuroplasticity and TrkB-mediated plasticity signaling [9], a molecular pathway through which psilocybin is proposed to exert lasting antidepressant effects. Unbiased high-throughput proteomic profiling using the Olink Target 96 Metabolism panel was employed to characterize treatment-associated changes in the circulating proteome enabling identification of novel signatures beyond the scope of targeted assays. Finally, measurement of p11 (S100A10) in peripheral blood mononuclear cells (PBMCs) was included since it has been implicated in MDD and antidepressant response to citalopram in MDD patients [10].

## Materials and methods

The study cohort has been described previously [6]. In brief, 35 participants were randomized to psilocybin or niacin. Samples were collected 1–2 days before dosing, and study medication was administered with 200 mL of water. CSF sampling was performed through lumbar punctures at the L3-L4 or L4-L5 level with the subject in an upright position. 5 ml of CSF was sampled and immediately centrifugated. The fluid was then divided into 1 ml aliquots and frozen until analyzed. Participants were monitored by psychologists, with a physician available if needed. Follow-up sampling was conducted approximately 14 days later. Seventeen participants in each group provided paired blood samples. For CSF, paired samples were obtained from 14 psilocybin-treated and 11 placebo participants. Only individuals with paired samples were included in statistical analyses.

For PBMC analyses, the psilocybin group had a mean age of 40.3 years, Montgomery–Åsberg Depression Rating Scale (MADRS) 25.9, and a 10:7female-to-male ratio; the placebo group had a mean age of 44.0 years, MADRS 26.1, and a ratio of 10:7. For CSF analyses, the psilocybin group had a mean age of 38.2 years, MADRS 25.5, and a ratio of 9:5, while the placebo group had a mean age of 47.2 years, MADRS 25.8, and a ratio of 8:3. The study was approved by the Swedish Ethical Review Authority and the Swedish Medical Products Agency. All participants provided written informed consent.

Detailed description of all procedures can be found in the supplementary information.

## Results

**Fig. 1:**
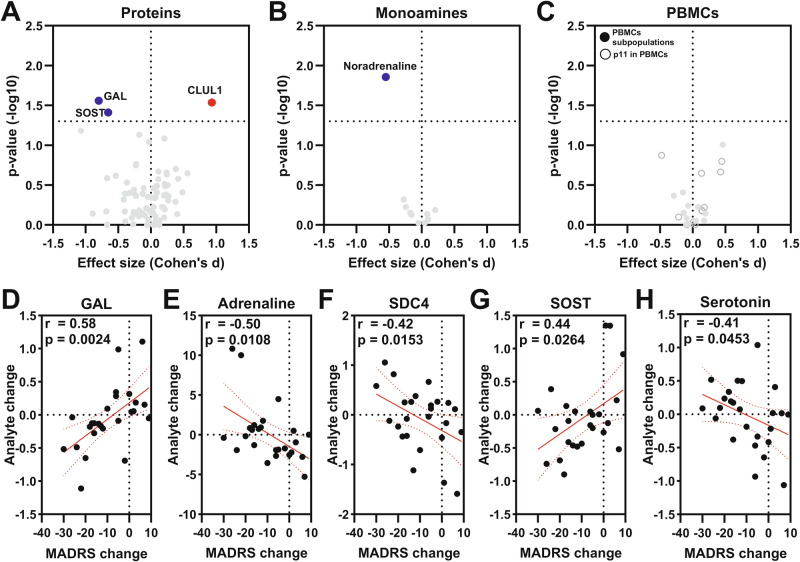
Biochemical changes induced by psilocybin versus placebo in patients with MDD. Between-group comparisons at follow-up are presented for CSF proteins (**A**), CSF monoamines (**B**), and p11 levels in PBMCs (open circle) and PBMC subpopulations (filled circle) (**C**). Correlation between MADRS score change from baseline to the post-treatment visit and change in galanin (**D**), adrenaline (**E**), SDC4 (**F**), SOST (**G**), and serotonin (**H**) levels. SDC4 Syndecan‑4, CLUL1 Clusterin‑like protein 1, GAL Galanin, SOST Sclerostin.

The changes in analyte levels between the placebo (niacin) and psilocybin-treated groups at the follow-up visit were analyzed using baseline levels as a covariate. In CSF protein analyses, combining a targeted cytokine panel (IL-1β, IL-6, TNF-α, IFN-γ) with the Olink Target 96 Metabolism panel, the psilocybin group exhibited reduced levels of galanin (GAL) and SOST, alongside increased levels of CLUL1 (Fig. 1A). Monoamine analysis revealed decreased noradrenaline levels in the psilocybin group (Fig. 1B). No differences were observed in p11 levels in PBMCs or across leukocyte subpopulations (Fig. 1C). CSF proBDNF/BDNF showed large variability with no significant changes. Finally, we assessed whether changes in analyte levels from baseline to the post-treatment visit correlated with changes in the MADRS. The strongest correlations were observed for galanin (Fig. 1D), followed by adrenaline (Fig. 1E), SDC4 (Fig. 1F), SOST (Fig. 1G), and serotonin (Fig. 1H). Both galanin and SOST were positively correlated with changes in MADRS scores, whereas adrenaline, serotonin, and SDC4 were negatively correlated with MADRS change.

## Discussion

This multi-modal biomarker study identified CSF alterations by psilocybin-assisted therapy in MDD. The most robust finding was a marked reduction of CSF galanin in the psilocybin group, both within-group and versus placebo at follow-up, and strong correlation between galanin and the MADRS score change from baseline to post-treatment visit. Galanin is a 29–30 amino acid neuropeptide, co-localized with noradrenaline in locus coeruleus (LC) neurons and serotonin in dorsal raphe neurons [11] and previously associated with MDD [12]. Galanin acts as a stress-inducible inhibitor of monoaminergic firing via Gi/o-coupled GALR1 and GALR3 receptors [13]. In MDD, post-mortem data show increased galanin and GALR3 expression in these regions with reduced DNA methylation, consistent with enhanced galaninergic inhibition of monoaminergic output [14]; genetic studies further link galanin polymorphisms to MDD risk [15]. The observed downregulation of CSF galanin after psilocybin aligns with this pathophysiological model.

The concurrent reduction of CSF NA, the only monoamine change observed, and galanin is unlikely to be coincidental. In the LC, galanin and noradrenaline are co-released, whereby galanin may inhibit LC firing via GALR1/3 autoreceptors and reduce noradrenaline release into downstream projection targets and may promote anxiety like behavior over the course of hours to days. However, an apparent paradox arises: if psilocybin reduces galanin, one might expect disinhibition of noradrenaline release and thus elevated rather than reduced CSF NA. Several mechanisms may explain parallel decreases. Psilocybin’s 5-HT2A agonism can directly suppress LC activity, lowering noradrenaline independently of galanin. Alternatively, both reductions may reflect normalization of LC hyperactivity in MDD, where galanin and noradrenaline are elevated. Consistent with this, antidepressants that reduce LC activity also decrease galanin expression, suggesting galanin tracks LC activity rather than simply opposing it.

Beyond galanin, the proteomics analysis identified significant changes in SOST and CLUL1 and trends in downregulation of SEMA3F and NT-proBNP. These proteins are involved in diverse processes spanning Wnt‑mediated tissue remodeling and osteokine signaling (SOST), cellular stress and metabolic regulation (CLUL1), axonal guidance and synaptic circuit refinement (SEMA3F), and cardiometabolic and vascular homeostasis (NT‑proBNP). None of them has previously been associated with MDD, psilocybin, or niacin. Their coordinated change suggests a shift toward a less stressed, less immune-activated, and less vascularly strained brain state. This pattern is consistent with psilocybin’s neuronal network resetting effects.

A notable aspect of the present study is the lack of significant psilocybin-induced changes in cytokines (IL-1β, IL-6, TNF-α, IFN-γ) and BDNF. Although prior work in healthy volunteers showed acute reductions in TNF-α and sustained decreases in IL-6 and CRP [8], the discrepancy with this MDD cohort may involve several factors. Timing of sample collection is critical, as cytokine effects appear stronger within the first week; later sampling, as done here, may miss these changes.

p11 was measured in PBMC subsets because decreases in NK cell and monocyte p11 have been associated with subsequent antidepressant response to chronic citalopram [10]. The absence of a p11 effect in the psilocybin group may reflect mechanistic differences from SSRIs, which directly elevate synaptic serotonin and may more strongly recruit p11-dependent serotonin receptor trafficking than a rapidly acting 5-HT2A agonist. p11 regulation by rapid-acting agents may also follow a different time course than with chronic SSRIs, so a relevant change could have occurred outside our sampling window. Accordingly, the null p11 finding here should be viewed not as evidence against a role for p11 in psilocybin’s mechanism, but as a constraint that can refine hypotheses and timing for future studies.

Several limitations should be acknowledged. Although the cohort size was sufficient for meeting primary clinical endpoints, it may have been underpowered to detect modest effects in secondary biomarker analyses. Biosampling timing is a key variable: psilocin has a ~ 2–3 h half-life, and while neuroplastic effects persist, optimal sampling windows for specific molecular readouts likely differ and cannot be captured at a single time point. Clinical heterogeneity within the cohort (MDD severity, treatment history, inflammatory subtype) may have diluted effects that could be evident in more homogeneous subgroups.

In conclusion, this study provides a neurochemical and proteomics characterization of psilocybin’s effects on CSF in MDD, identifying downregulation of the galanin- noradrenaline axis as a key pharmacodynamic signature. Reduced CSF galanin emerges as a novel, biomarker of antidepressant effect that warrants validation in larger cohorts and further correlation with clinical outcomes. These findings refine the mechanistic framework for psilocybin therapy and suggest the galanin system as a potential mediator of its sustained neurobiological effects.

## Supplementary information

Supplementary Methods and Results

## Data Availability

De-identified individual participant data underlying the findings reported in this study may be made available by the corresponding author upon reasonable request, subject to the absence of legal, regulatory, or ethical restrictions.
