# Supplementary material for: CSF galanin and noradrenaline downregulation by psilocybin therapy in major depressive disorder
Source: Neuropsychopharmacology. 2026 Jul 7;51(10):1898–901. doi: 10.1038/s41386-026-02490-3 (PMC13487249; doi:10.1038/s41386-026-02490-3)
Supplement: Supplementary file 1 [file 41386_2026_2490_MOESM1_ESM.docx]

**Supplementary information for**

**CSF Galanin and Noradrenaline Downregulation by Psilocybin Therapy in Major Depressive Disorder**

Wojciech Paslawski^1^, Daniel Doyon^1^, Carl Johan Ekman^2^, Hampus Yngwe^2^, Dejan Mamula^1^, Justyna Zareba-Paslawska^1^, Maria Beckman^2^, Zhi-Qing David Xu^3^, Tomas Hökfelt^4^, Mikael Tiger^2^, Johan Lundberg^2§^, Per Svenningsson^1*§^

^1^Translational Neuropharmacology, Department of Clinical Neuroscience, Karolinska Institutet, Stockholm, Sweden

^2^Centre for Psychiatry Research, Department of Clinical Neuroscience, Karolinska Institutet, and Stockholm Health Care Services, Region Stockholm, Stockholm, Sweden

^3^Departments of Neurobiology and Pathology, Capital Medical University, Beijing, China

^4^Department of Neuroscience, Karolinska Institutet, Stockholm, Sweden

*Corresponding author

^§^Shared last authorship

**Supplementary Materials and Methods**

***Chemicals:*** Unless stated otherwise, all chemicals were purchased from Sigma-Aldrich (Merck KGaA) and were of an analytical grade. All solutions were prepared using Milli-Q deionized water (Millipore).

***Proximity extension assay (PEA):*** Multiplex PEA was performed using Olink platform as described before^1^. All samples were run on the Metabolism 96-plex panel. The list of proteins and their validation data are freely available online (https://www.olink.com/data-you-can-trust/validation/). The data for each protein is given as a normalised protein expression (NPX) value, an arbitrary unit on a Log2 scale and normalised to minimise both intra-assay and inter-assay variations.

**Analysis of neurotransmitters by high-performance liquid chromatography (HPLC):** HPLC wase based on previously published protocols^2,3^. Briefly, ice-cold perchloric acid was added to CSF sample, incubated on ice for 10 minutes, vortexed and centrifuged at 12000 x g for 10 minutes at 4°C. Resulting supernatants were filtered through 0.2 µm nylon membrane inserts. Eluents were immediately stored at -80°C and subjected to HPLC analysis within 1 week. Standard solutions of: L-noradrenaline hydrochlorid, (+/-)-Adrenaline hydrochloride, 3,4-dihydroxyphenylacetic acid, 3,4-Dihydroxy-L-phenylalanine, dopamine hydrochloride, 5-hydroxyindole-3-acetic acid, homovanillic acid, serotonin hydrochloride, 4-Hydroxy-3-methoxyphenylglycol hemipiperazinium salt, DL-4-Hydroxy-3-methoxymandelic acid and 3-Methoxytyramine hydrochloride were used for calibration curves. The HPLC system used was a Dionex Ultimate 3000 series with Chromeleon software (Dionex, ThermoFisher Scientific, USA). Analyte separation was performed on a Dionex C18 reversed-phase MD-150 3.2 mm x 250 mm column. Column and analytical cell were kept at 30 °C. The mobile phase consisted of 75 mM monobasic sodium phosphate, 2.2 mM 1-octanesulfonic acid sodium salt, 100 µL/L triethylamine, 25 µM ethylene-diamine-tetra-acetic acid disodium salt and 10 % acetonitrile (v/v), pH 3.0 adjusted with 85% phosphoric acid. The first and second analytical cells were set to -300 mV and +300 mV, respectively. Analyte concentrations in CSF were expressed as ng/mL of CSF.

***Cytokine and Neurotrophin Quantification:*** Concentrations of BDNF, IFN-γ, IL-1β, IL-6, and TNF-α were quantified in CSF using commercially available immunoassay platforms. Mature BDNF and proBDNF were measured simultaneously using the Mature BDNF/proBDNF Combo Rapid ELISA Kit (BEK-2240-2P, Biosensis; distributed by VWR) following the manufacturer's protocol. The pro-inflammatory cytokines IFN-γ, IL-1β, IL-6, and TNF-α were quantified in parallel using the V-PLEX Human Proinflammatory Panel I multiplex electrochemiluminescence immunoassay (K15052D1, Meso Scale Discovery, MSD), with all steps performed in accordance with the manufacturer's instructions.

***Flow cytometry:*** Flow cytometry of patient PBMCs was performed based on previously published protocols^4,5^. In brief, frozen PBMC were thawed quickly and immediately washed with PBS. Leucocytes were then stained with live/dead fixable violet dead cell marker (Invitrogen, cat. nr. L10119) for viability staining. After washing with PBS, cells were fixed and permeabilized with BD Cytofix/Cytoperm fixation and permeabilization solution (BD, cat. nr. 554714) for 20 min, then stained with monoclonal mouse anti-human annexin II light chain (i.e. p11) antibody (5μg/mL, clone 148, BD, cat. nr. 610071) for 45min at RT. Secondary staining was performed using APC-conjugated goat anti-mouse antibody (2 μg/mL, BioLegend, cat. nr. 405308) for 30min at RT. Cells were washed twice with BD permeabilization buffer (cat. nr. 561651), then blocked in PBS with 1%FBS and 1%NMS for 20min. Cells were then stained a mixture of surface antibody markers to identify cytotoxic CD8+ T cells and CD4+ T helper cells, B lymphocytes (CD19+), classically activated monocytes (CD3-CD14+CD16-), non-classically activated monocytes (CD3-CD14-CD16+) and NK cells (CD3-CD16+CD56+): CD3 PerCP/Cy5.5 clone UCHT1, CD4 PE/Cy7 clone OKT4, CD8 BV510 clone SK1, CD14 APC/Cy7 clone MφP9, CD16 PE clone 3G8, CD19 BV605 clone HIB19 and CD56 FITC clone B159. After washing twice with PBS, cells were analyzed using the flow cytometer Sony ID7000 spectral analyzer. Data were collected and analyzed using FlowJo 10.10.0 (BD). P11 levels in each cell population were determined using the appropriate gating strategy and p11 median fluorescence intensity (MFI) was used. The p11 antibody demonstrated high specificity across all analyzed cell subsets, which was confirmed using an isotype control antibody, as previously reported by our group^4-6^.

***Statistical analyses:*** The statistical analyses were performed using RStudio and GraphPad Prism. For PEA assay proteins with > 30% values falling under the limit of detection (LOD) were excluded from analyses. The data were analyzed using an ANCOVA mixed model, with follow-up values as the outcome, baseline as a covariate, and treatment group as a fixed factor. In this framework, the group × time interaction is captured by the treatment group effect on baseline-adjusted follow-up values. Associations between datasets were assessed using Spearman correlation. The p-values were adjusted for multiple comparisons using the Benjamini–Krieger–Yekutieli (BKY) false discovery rate method. *P*-values < 0.05 were considered as statistically significant.

**Supplementary results**

As stated in main manuscript CSF proBDNF/BDNF showed large variability with no significant changes. The group characteristics were as follows.

**ProBDNF:**

Psylocibin baseline – mean 4.0 pg/mL, median 0.0 pg/mL, standard deviation 11.6 pg/mL, range 0-43.6

Placebo baseline – mean 17.8 pg/mL, median 0.0 pg/mL, standard deviation 49.2 pg/mL, range 0-165.6

Psylocibin after treatment – mean 1.3 pg/mL, median 0.0 pg/mL, standard deviation 3.6 pg/mL, range 0-12.4

Placebo after treatment – mean 19.5 pg/mL, median 0.8 pg/mL, standard deviation 51.5 pg/mL, range 0-173.3

Baseline psilocybin vs. placebo p-value 0.32

After treatment psilocybin vs. placebo p-value 0.28

Psilocybin baseline vs after treatment	p-value 0.22

Placebo baseline vs after treatment p-value 0.48

**BDNF:**

Psylocibin baseline – mean 5.3 pg/mL, median 0.9 pg/mL, standard deviation 16.6 pg/mL, range 0-63.1

Placebo baseline – mean 7.0 pg/mL, median 2.1 pg/mL, standard deviation 14.2 pg/mL, range 0-48.6

Psylocibin after treatment – mean 1.0 pg/mL, median 0.4 pg/mL, standard deviation 1.2 pg/mL, range 0-4.1

Placebo after treatment – mean 6.6 pg/mL, median 0.7 pg/mL, standard deviation 16.3 pg/mL, range 0-55.0

Baseline psilocybin vs. placebo p-value 0.75

After treatment psilocybin vs. placebo p-value 0.31

Psilocybin baseline vs after treatment	p-value 0.24

Placebo baseline vs after treatment p-value 0.90

**Supplementary references**

1	Paslawski, W. *et al.* Large-scale proximity extension assay reveals CSF midkine and DOPA decarboxylase as supportive diagnostic biomarkers for Parkinson's disease. *Translational neurodegeneration* **12**, 42, doi:10.1186/s40035-023-00374-w (2023).

2	Sousa, V. C. *et al.* P11 deficiency increases stress reactivity along with HPA axis and autonomic hyperresponsiveness. *Molecular psychiatry* **26**, 3253-3265, doi:10.1038/s41380-020-00887-0 (2021).

3	Shariatgorji, R. *et al.* Quantitative Mass Spectrometry Imaging Protocols for Spatially Heterogeneous Samples. *Anal Chem* **97**, 10957-10961, doi:10.1021/acs.analchem.5c00677 (2025).

4	Veldman, E. R. *et al.* P11 (S100A10) as a potential predictor of ketamine response in patients with SSRI-resistant depression. *Journal of affective disorders* **290**, 240-244, doi:10.1016/j.jad.2021.04.055 (2021).

5	Green, H. *et al.* Alterations of p11 in brain tissue and peripheral blood leukocytes in Parkinson’s disease. *Proceedings of the National Academy of Sciences* **114**, 2735-2740, doi:10.1073/pnas.1621218114 (2017).

6	Mamula, D., Khosousi, S., He, Y., Lazarevic, V. & Svenningsson, P. Impaired migratory phenotype of CD4(+) T cells in Parkinson's disease. *NPJ Parkinsons Dis* **8**, 171, doi:10.1038/s41531-022-00438-0 (2022).
